# Characterizing Highly Frequent Users of a Large Canadian Urban Emergency Department

**DOI:** 10.5811/westjem.2018.9.39369

**Published:** 2018-10-18

**Authors:** Julie J. Kim, Edmund S.H. Kwok, Olivia G. Cook, Lisa A. Calder

**Affiliations:** * University of Ottawa, Department of Emergency Medicine, Ottawa, Ontario, Canada; † Ottawa Hospital Research Institute, Ottawa, Ontario, Canada; ‡ University of Ottawa, Department of Undergraduate Medicine, Ottawa, Ontario, Canada

## Abstract

**Introduction:**

Highly frequent users (HFU) of the emergency department (ED) are a poorly defined population. This study describes patient and visit characteristics for Canadian ED HFU and patient subgroups with mental illness, substance misuse, or ≥ 30 yearly ED visits.

**Methods:**

We reviewed health records from a random selection of adult patients whose visit frequency comprised the 99th percentile of yearly ED visits to The Ottawa Hospital. We excluded scheduled repeat ED assessments. We collected the following: 1) patient characteristics – age, sex, and comorbidities; and 2) ED visit characteristics – diagnosis category, length of stay, presentation time, consultation services, and final disposition. Two reviewers collected data, and we performed an inter-rater review to measure agreement.

**Results:**

We analyzed 3,164 ED visits for 261 patients in all subgroups overall. Within the HFU random selection, mean age was 53.4 ± 1.3, and 55.6% were female. Most patients had a fixed address (88.9%), and family physician (87.2%). Top ED diagnoses included musculoskeletal pain (9.6%), alcohol intoxication (8.5%), and abdominal pain (8.4%). Allied health (social work, geriatric emergency medicine, or community care access centre) was consulted for 5.9% of visits. In 52.7% of these cases, allied health services were not available at the time of presentation.

**Conclusion:**

HFU are a complex population who represent a marked proportion of annual ED visits. Our data indicate that there are opportunities to improve the current approaches to care. Future work examining ED-based screening and multi-disciplinary approaches for HFU may help reduce frequent ED presentations, and better serve this vulnerable population.

## INTRODUCTION

Highly frequent users (HFU) of the emergency department (ED) are a poorly defined population. A systematic review of frequent ED users in the United States suggested that this group comprised only 4.5–8% of ED patients, but accounted for up to 21–28% of all ED visits.^1^ The Canadian literature is sparse, and to date there is a lack of a clear definition of HFU in urban academic centres.^2^,^3^ Systematic reviews including international and Canadian studies have included definitions ranging from 3–20 ED visits per year.^1^,^2^ The limited number of Canadian studies and lack of consistent HFU definition is an issue for healthcare providers and communities that aim to improve the quality of healthcare and reduce frequent ED use.^3^,^4^

HFU have been described as a heterogeneous population, with patient presentations for both significant medical and social reasons.^5^–^9^ As such, pre-existing attempts to address the needs of these patients to reduce ED presentation have had mixed success in the literature.^10^,^11^ The HFU population has an increased prevalence of chronic disease, and mental health and substance misuse issues.^2^ These attributes suggest a need for further focus on these subgroups. The objectives of this study were first to examine HFU within a Canadian urban academic ED based on a distribution cutoff of the 99^th^ percentile of ED visits, and second to further characterize subgroups with substance misuse and mental illness issues within this population.

## METHODS

We conducted a health records review of patients whose visit frequency comprised the 99^th^ percentile of ED visits to the Ottawa Hospital between January 1 and December 31, 2014. The Ottawa Hospital is a large Canadian urban academic teaching centre, comprised of multiple campuses, which includes two EDs that received over 140,000 ED visits at the time of this study. The Ottawa Hospital is the regional trauma centre with high volumes of cardiac, dialysis, neurosurgical and cancer patients for the city and surrounding area. Ethics approval for this study was granted by the Ottawa Health Science Network Research Ethics Board.

### Data Source and Patient Selection

We used The Ottawa Hospital Data Warehouse, a database with operational and patient information for research and quality assurance purposes, to identify eligible patients. Eligible patients were 18 years or older, whose ED visit frequency was greater than a distribution cutoff greater than the 99^th^ percentile of ED visits, which was a minimum of seven times in 2014. Applying a standard definition using the 99^th^ percentile captures the greatest outliers in patients who frequent the ED, while proportionally reflecting the volume and frequency of patients seen at our centre on an annual basis. We excluded visits for scheduled repeat assessments in the ED. We used a computerized random sample generator to select 250 patients evenly distributed by number of presentations (i.e., 7, 8, 9, 10, and 11 or more visits that year). We extracted patient and visit characteristic data from the ED record of treatment, nursing notes, and consultant notes from each visit. We collected the following patient characteristics: age at first ED visit that year, sex, medical comorbidities, listed family physician, and documentation of a fixed address. We characterized comorbidities by body systems and associated risk factors (i.e., cardiac disease) rather than specific comorbidity, due to the extensive range of comorbid conditions among ED patients. For example, cardiac comorbidities included a history of myocardial infarction, angina, hypertension and/or dyslipidemia.

We included the following visit characteristics: Canadian Triage Acuity Scale (CTAS) score,^12^ ED time of arrival, ED length of stay (LOS), ED discharge diagnosis category, consultations made, and disposition from the ED. The CTAS is a validated triage system that prioritizes patient care by severity of illness and assigns a recommended time to patient initial assessment.^14^ For example, CTAS 1 (resuscitation) patients should be seen immediately, CTAS 2 (emergency) within 15 minutes, CTAS 3 (urgent) within 30 minutes, CTAS 4 (less urgent) within 60 minutes, and CTAS 5 (non-urgent) within 120 minutes. We collected ED discharge diagnoses as documented on patient health records, and similar diagnoses were later grouped into appropriate categories for reporting purposes. For example, acute myocardial infarction and acute coronary syndromes were grouped into chest pain, whereas non-cardiac chest pain and chest wall pain were grouped into musculoskeletal chest pain. See Appendix 1 for full list of ED discharge diagnosis categories. Two reviewers (JK, OC) manually reviewed all ED records of treatment, which are hand-written but electronically scanned. We reviewed specialist consultant notes for ED visits on an as-needed basis for clarification of diagnosis, disposition, or patient comorbidities. We performed inter-rater review of randomly abstracted patient visits at two periods early within data collection.

**Population Health Research Capsule**

What do we already know about this issue?

Highly frequent users (HFU) of the Emergency Department (ED) are a poorly defined population, with an increased prevalence of chronic disease, mental health, and substance misuse.

What was the research question?

We examined HFU using a 99th percentile cutoff, and characterized subgroups with history of substance misuse and mental illness.

What was the major finding of the study?

Top diagnoses included painful conditions and alcohol-related visits. Allied health consultants were often unavailable.

How does this improve population health?

Our data highlight discrepancies between the nature of HFU visits and the availability of acute care resources to serve medical and social needs of this complex population.

### Analysis

We conducted our analyses using *SAS version 9.3* software (SAS Institute, Cary, NC) and performed descriptive and univariate analyses. We compared frequencies using chi-squared and Student’s t-tests for normally distributed data. ED LOS was not normally distributed, and thus was analyzed by Mann-Whitney U tests for non-parametric distributions. To ensure adequate inter-rater reliability and consistency of the health records review, we used Cohen’s kappa to measure levels of agreement for categorical variables early in data collection. We performed subgroup analyses for patients with a documented history of mental illness or substance misuse. All patients who had ≥ 30 ED visits in 2014 were also included for additional subgroup analyses, if not already selected randomly. Patients who presented ≥ 30 times composed the most frequent 2% of all HFU who were eligible for inclusion.

## RESULTS

Between January 1 to December 31 of 2014, 93,762 patients visited the Ottawa Hospital EDs on 140,503 separate occasions. The majority of these patients (95.2%) visited the ED on 1–3 occasions, which accounted for 81.5% of yearly ED visits. There was a smaller subset of frequent users who visited the ED 4–6 times, comprising 3.9% of the yearly ED patients and 11.8% of ED visits. The HFU who presented a minimum of seven times (> 99^th^ percentile of ED visits) totaled 897 patients with 9,376 visits. As per our study definition, our HFU consisted of the most frequent 1.0% of ED patients, and comprised 6.7% of yearly ED visits. The maximum number of ED visits by a single patient that year was 84 separate visits.

**Figure 1:**
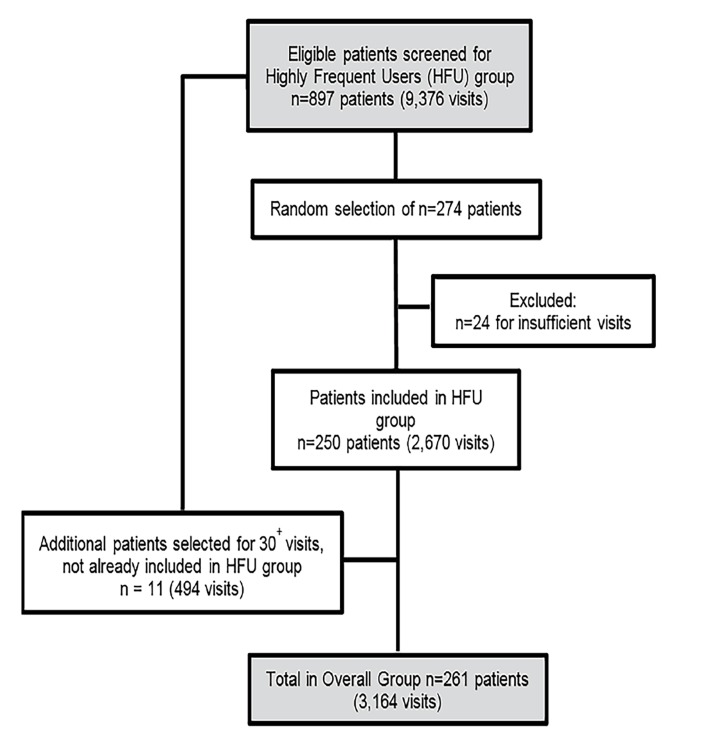
Patient selection process for highly frequent users of the emergency department.

The random selection of HFU resulted in 2,670 ED visits, and totaled 3,164 ED visits when including all subgroups (Figure 1). We excluded 24 patients for insufficient visits. These patients would have been included automatically by the Data Warehouse database for visiting the ED a minimum of seven times in the year. However, if a patient was seen directly by a consulting service and not the emergency physician, this would have excluded them from the minimum number of seven presentations to qualify as a HFU. The characteristics of patients and their comorbidity type listed by system category are identified in Table 1. The majority of patients had a family physician and fixed address at the time of their ED visit. The greatest percentage of patient comorbidity type included gastrointestinal problems, cardiac diagnoses or risk factors, and chronic pain. Alcohol was the most commonly misused substance, while anxiety and depression were the most commonly represented mental illnesses.

The characteristics of each ED visit by CTAS score, ED LOS, and disposition are listed in Table 2. The majority of patient visits (90.9%) had a CTAS score of 2 or 3, indicating acute presentations with a recommended physician assessment within 15 or 30 minutes respectively.^12^ Median ED LOS was 5.2 hours, with an inter-interquartile range (IQR) of 3.1–9.0 hours. Most HFU were discharged home or to an outside residence from the ED, but 15.6% of HFU visits required hospital admission, and 5.1% of visits from the 30^+^ subgroup required admission. Comparatively, the baseline proportion of hospital admissions from the ED during 2014 was 17.1%. The ED diagnoses were grouped into appropriate categories and are listed in Table 3. Abdominal pain, alcohol intoxication and musculoskeletal pain were within the top five diagnostic categories overall, and for each subgroup analyzed. Overdose or substance misuse aside from alcohol intoxication was in the top five ED diagnoses only for patients with a history of mental illness, substance misuse, or patients with 30^+^ visits.

**Figure 2:**
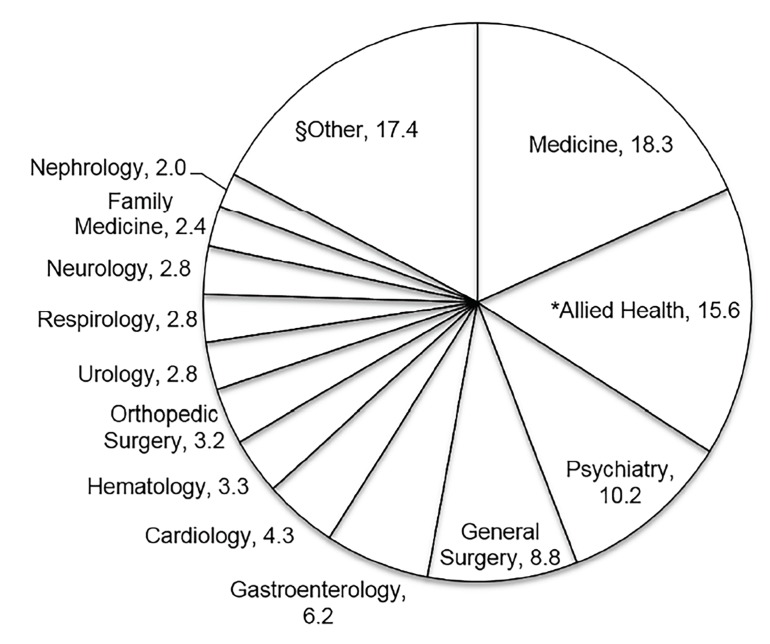
Proportion of consultations for highly frequent users. *Allied Health includes consultations for social work, community care access centre, and geriatric emergency medicine nurses combined. ^§^Other indicates all other services consulted from the emergency department not listed above, and individually <2% of consultations. n=261 patients and 3,164 visits.

Specialist services that received the most consultations for HFU are shown in Figure 2. Internal medicine received the most consultations (18.3%), followed by psychiatry (10.2%), and social work (10.1%). Our allied health consultants who consist of social workers, geriatric emergency medicine (GEM) nurses, and community care access centre (CCAC) workers, received 15.6% of the HFU consultations altogether or 10.1%, 1.7% and 3.8% of consultations, respectively. CCAC is a community service that provides transitional home care for patients who may need additional assistance. This may include nursing support, physiotherapy, occupational therapy, social work support or medical supplies and equipment at home. For example, services may include daily dressing changes from a wound care nurse, administration of intravenous antibiotics at home, mobility support from a physiotherapist, or a home safety assessment by an occupational therapist. Overall, our allied health consultants provided support for 5.9% of ED visits for the HFU population. Within the “*Other”* consultant category, the most consulted specialists included infectious disease (1.9% of consults), psychiatric emergency services (psychiatric nurses and/or social workers but not psychiatrists) (1.8%), obstetrics and gynecology (1.8%), and medical oncology (1.8%).

**Figure 3:**
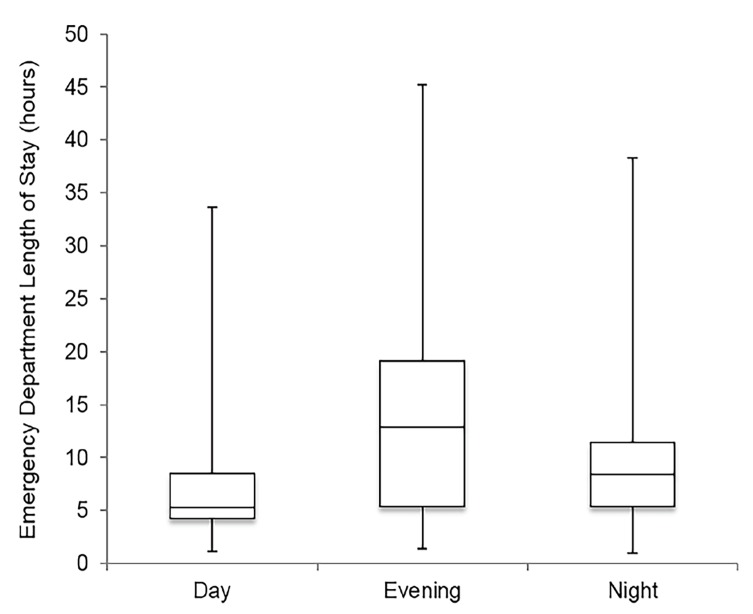
Emergency department length of stay by time of presentation. Box and whisker plots representing median emergency department length of stay with inter-quartile ranges for n=3,164 visits. Day: 0800-1559 hours (h); Evening: 1600-2359 h; Night: 0000-0759 h.

**Figure 4:**
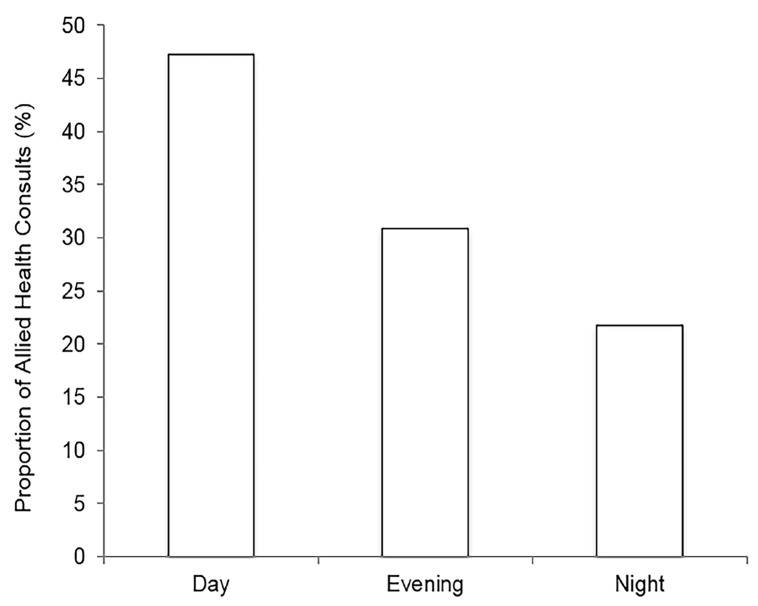
Proportion of Allied Health consults by time of presentation. n=203 allied health consultations during 188 of 3164 possible visits. Allied health consultants included social work, geriatric emergency medicine nurses, or community care access workers. Day: 0800-1559 hours (h); Evening: 1600-2359 h; Night: 0000-0759 h.

Overall, roughly two thirds of ED presentations were between 4 pm – 7:59 am, outside of daytime hours. The subset of patients with 30^+^ visits had a slightly higher proportion of visits (67%) outside of daytime hours. Figure 3 illustrates ED LOS stratified by time of ED presentation. As shown by the box and whisker plots, median ED LOS was significantly higher in the evening (12.7 hours, range 1.4–45.2 hours) compared to the daytime (5.4, 1.2–33.6; p=0.0002) as well as night (7.9, 1.0–38.3, p=0.02). Figure 4 depicts the proportion of allied health consultations and corresponding time of patient presentation to the ED. Bars show that 47.3% of consultations were made during the day, while 52.7% were made in the evening and night, 30.9% and 21.8% respectively.

To ensure adequate inter-rater reliability and consistency of the health records review, we examined 4.5% of abstracted health records (142 patient visits with 4,515 variables) early in data collection to reveal a Cohen’s kappa score of 0.8 for agreement between our two reviewers (JK, OC).

## DISCUSSION

This is the first study to analyze HFU of a large urban ED in Canada, using a well-defined distribution-based percentile cutoff as opposed to an absolute cutoff in number of visits. This method was first described in a smaller suburban setting,^13^ and by using a statistical threshold rather than an absolute number of visits, it can be reproducibly applied to large or small EDs regardless of volume variations. Our results reflect that HFU are a heterogeneous and complex population.

Several patterns emerged from this analysis. The ED discharge diagnoses of HFU groups and subgroups analyzed in our study consistently highlighted an abundance of alcohol- and pain-related visits. There also appeared to be a discrepancy between the needs of HFU and the availability of allied healthcare support depending on time of presentation to the ED. While the majority of patients who received allied health consultations arrived in the evening or night, they were required to wait in the ED for a consultation in the morning when the service became available. This was reflected in a significantly prolonged ED LOS. It is important to note that social workers, GEM nursing and CCAC consultations are not available at our site for the majority of evening or night time hours, whereas most other consultant specialties are available 24 hours a day, 7 days a week.

While lack of access to a family physician was previously thought to be a strong predictor for frequent ED visits, studies have now suggested that many patients who frequent the ED do have family physicians.^14^,^15^ We identified that 87.2% of HFU in our study had a family physician, suggesting that access to a family physician may not be sufficient to address the needs of this population or reduce frequent ED visits. In 2016, 84.2% of those aged 12 or older in Canada reported having a regular healthcare provider, and males who were 18–34 were more likely than any other group to be without a family physician.^16^ Of the 15.2% without a regular healthcare provider, the most commonly reported reasons were that they “had not tried to find one” or “did not need one” (28.7%). In the province of Ontario in 2016, 94.3% of Canadians aged 16 or older reported having a primary physician.^17^ Same-day response to phone calls to a primary care office in 2016 were 78.9%, but availability of same-day or next-day appointments was only 43.1%.^17^ While primary group practices are beginning to offer patients after-hour clinics, ED-based screening and proactive interventions aimed at understanding and modifying other barriers to primary or outpatient healthcare access for HFU may better serve to address frequent ED presentation.

Research is now beginning to focus on quality improvement strategies for coordination of outpatient care for ED patients with chronic conditions. Evidence is emerging that interventions such as dedicated case management can reduce ED use and associated healthcare costs for this population.^4^,^18^–^21^ Case management was noted to significantly reduce identified issues such as homelessness, alcohol misuse, and financial need.^19^,^22^ However, reviews of these strategies suggest the need for further research to determine the specific aspects of case management that are most successful and effective in reducing ED visits in frequent users.^10^,^23^,^24^

## LIMITATIONS

While our study was able to capture patient and visit data in much more detail than is possible for typical administrative database studies, the following limitations should be considered. By reviewing individual charts, we were able to review many visits, but only a relatively small number of patients. We relied on the legibility of physician handwriting, which was highly variable. We used consultant notes to capture patient comorbidities and past medical history when hand-written emergency charts were illegible. This may have contributed to an underestimation of patient comorbidities if only the main or contributing comorbidities to the visit were listed on the record. We recognize that the chart abstracters were not blinded to the objectives of the study. In addition, there may be limitations in the generalizability of this study as a single urban site in Canada, noting that variability in patient comorbidities, social needs, and available services may exist based upon geographic location. Finally, we examined ED visits to our study sites without access to data from surrounding EDs in the city. Patients may have visited other EDs in the region, but our previous research suggests this is rare.^25^,^26^

## CONCLUSION

HFU are a complex population who represent a marked proportion of annual ED visits, and our data indicate that there are opportunities to improve current approaches to their care. We have highlighted the discrepancy between the social needs of these patients and the availability of allied health resources when many HFU present to the ED. Our data suggest a need for more than emergency or primary management of chronically complex patients in an acute care setting such as the ED. Future work examining proactive screening for outpatient programs in chronic pain and substance misuse may help reduce frequent ED presentations, and better serve patients with complex medical and social needs.

## Supplementary Material



## Figures and Tables

**Table 1:** Number and (percentage) of patients unless otherwise indicated.

Variable	Number (%) of patients				

	Overall	Highly frequent users	Substance misuse history	Mental illness history	30^+^ Visits

	n = 261	n = 250	n = 77	n = 107	n = 18
Age (in years)					
Mean ± SEM	52.7 ± 1.3	53.4 ± 1.3	45.5 ± 1.8	48.7 ± 1.8	38.5 ± 3.8
Range	18 – 96	18 – 96	18 – 82	18 – 88	18 – 62
Female sex	147 (56.3)	139 (55.6)	35 (45.5)	68 (63.6)	13 (72.2)
Family physician	225 (86.2)	218 (87.2)	56 (72.7)	93 (86.9)	12 (66.7)
Fixed address	231 (88.5)	222 (88.8)	52 (67.5)	88 (82.2)	13 (72.2)
Comorbidity system category					
Respiratory	109 (41.8)	103 (41.2)	24 (31.2)	41 (38.3)	7 (38.9)
Cardiac*	132 (50.6)	130 (52)	32 (41.6)	47 (43.9)	5 (27.8)
Gastrointestinal	163 (62.5)	154 (61.6)	48 (62.3)	61 (57.0)	12 (66.7)
Genitourinary	103 (39.5)	99 (39.6)	18 (23.4)	30 (28.0)	6 (33.3)
Musculoskeletal and soft tissue	116 (44.4)	111 (44.4)	30 (39)	44 (41.1)	8 (44.4)
Chronic pain	126 (48.3)	118 (47.2)	29 (37.7)	48 (44.9)	13 (72.2)
Endocrine	85 (32.6)	83 (33.2)	19 (24.7)	39 (36.4)	4 (22.2)
Neurological	112 (42.9)	106 (42.4)	30 (39)	55 (51.4)	7 (38.9)
Other medical comorbidity	129 (49.4)	122 (48.8)	28 (36.4)	44 (41.1)	10 (55.6)
Substance misuse history	80 (30.7)	77 (30.8)		48 (44.9)	6 (33.3)
Alcohol	53 (20.3)	52 (20.8)	53 (68.8)	30 (28.0)	3 (16.7)
Intravenous drug use	14 (5.4)	13 (5.2)	13 (16.9)	11 (10.3)	2 (11.1)
Opioids	14 (5.4)	12 (4.8)	12 (15.6)	9 (8.4)	3 (16.7)
Marijuana	23 (8.8)	21 (8.4)	21 (27.3)	15 (14.0)	3 (16.7)
Other substance misuse	12 (4.6)	11 (4.4)	11 (14.3)	9 (8.4)	1 (5.6)
Mental illness history	117 (44.8)	107 (42.8)	49 (63.6)		14 (77.8)
Anxiety	58 (22.2)	53 (21.2)	22 (28.6)	53 (49.5)	8 (44.4)
Depression	71 (27.2)	63 (25.2)	32 (41.6)	63 (58.9)	11 (61.1)
Psychosis/schizophrenia	24 (9.2)	20 (8.0)	10 (13.0)	20 (18.7)	5 (27.8)
Bipolar disorder/mania	18 (6.9)	18 (7.2)	12 (15.6)	18 (16.8)	0 (0)
Personality disorder	20 (7.7)	17 (6.8)	10 (13.0)	17 (15.9)	5 (27.8)
Other mental illness	25 (9.6)	21 (8.4)	12 (15.6)	21 (19.6)	4 (22.2)

*SEM*, standard error of means.

* Cardiac category includes cardiac conditions and risk factors.

**Table 2:** Number and (percentage) of patients unless otherwise indicated.

Variable	Number (%) of visits	

	n = 2,670 visits	n = 3,164 visits
Canadian triage acuity scale		
1	14 (0.5)	17 (0.5)
2	1,049 (39.3)	1,243 (39.3)
3	1,377 (51.6)	1,633 (51.6)
4	208 (7.8)	240 (7.6)
5	22 (0.8)	31 (1.0)
Emergency department length of stay, median hours (IQR)	5.2 (3.1–9.0)	5.2 (3.1–8.7)
Disposition		
Home	1,764 (66.1)	2,051 (64.8)
Admission	417 (15.6)	451 (14.3)
Shelter	202 (7.6)	209 (6.6)
Retirement or nursing home	127 (4.8)	202 (6.4)
Group home	70 (2.6)	153 (4.8)
Home with supports	18 (0.7)	18 (0.6)
Left without being seen	19 (0.7)	21 (0.7)
Left against medical advice	18 (0.7)	20 (0.6)
Mobile crisis	7 (0.3)	7 (0.2)

*IQR*, interquartile range.

**Table 3:** Emergency department discharge diagnoses were grouped into appropriate categories.

Patient group or subgroup	Number (%) of visits
Overall visits n = 3,164	
Abdominal pain	329 (10.4)
Alcohol intoxication	227 (7.2)
Musculoskeletal pain	204 (6.4)
Genitourinary infection	111 (3.5)
Chest pain	84 (2.7)
Highly frequent users n = 2,670	
Musculoskeletal pain	256 (9.6)
Alcohol intoxication	227 (8.5)
Abdominal pain	223 (8.4)
Genitourinary infection	90 (3.4)
Chest pain	81 (3.0)
Substance misuse history n = 889	
Alcohol intoxication	227 (25.5)
Overdose or substance misuse	52 (5.8)
Musculoskeletal pain	49 (5.5)
Abdominal pain	41 (4.6)
Chest pain	32 (3.6)
Mental illness history n = 1,202	
Alcohol intoxication	101 (8.4)
Musculoskeletal pain	96 (8.0)
Abdominal pain	85 (7.1)
Overdose or substance misuse	53 (4.4)
Chest pain	50 (4.2)
30^+^ Visits n = 801	
Abdominal pain	190 (23.7)
Flank pain	81 (10.1)
Alcohol intoxication	48 (6.0)
Musculoskeletal pain	35 (4.4)
Overdose or substance misuse	34 (4.2)
