# Supplementary material for: Characterizing Highly Frequent Users of a Large Canadian Urban Emergency Department
Source: West J Emerg Med. 2018 Oct 18;19(6):926–33. doi: 10.5811/westjem.2018.9.39369 (PMC6225932; doi:10.5811/westjem.2018.9.39369)
Supplement: Supplementary file 1 [file wjem-19-926-s001.docx]

Supplementary Material

| **Appendix 1: List of ED Discharge Diagnoses** | |  |
| --- | --- | --- |
| Abdominal Pain | Folliculitis | Panic Disorder |
| Abnormal Lab Values | Foreign Body | Paranoid Ideation |
| Abscess | Form 1 | Parasite |
| Acute Kidney Injury | Fracture | Paresthesias |
| Acute Myeloid Leukemia | Frostbite | PEG Tube Issue |
| Adjustment Disorder | Gallstones | Pelvic Infectious Disease |
| Akathisia | Gastric Obstruction | Pelvic Pain |
| Alcohol Intoxication | Gastritis | Penile Redness |
| Alcohol Withdrawl | Gastroenteritis | Peptic Ulcer Disease |
| Allergic Reaction | Gastroesophageal Reflux | Percutaneous Drain Removal |
| Amaurosis Fugax | Gastrointestinal Bleed | Perforated Bowel |
| Anaphylaxis | Gastroparesis | Perianal Abscess |
| Anemia | Globe Rupture | Perianal Fissure |
| Anorexia | Gout | Perineal Abscess |
| Anxiety | Hallucinations | Periorbital Cellulitis |
| Appendicitis | Head Injury | Peripheral Vascular Disease |
| Arterial Occlusion | Headache | Peripheral Vertigo |
| Ascites | Heel Ulcer | Pharyngitis |
| Assault | Hematemesis | PICC Issue |
| Asthenia | Hematochezia | Pleural Effusion |
| Asthma | Hematuria | Pneumonia |
| Atalectasis | Hemolysis | Pneumothorax |
| Atrial Fibrillation | Hemoptysis | Post Exposure Prophylaxis |
| Atrial Flutter | Hemorrhagic Cyst | Post Herpetic Neuralgia |
| Baker's Cyst | Hemorrhoids | Post Operative Complication |
| Biliary Colic | Hepatic Encephalopathy | Post Phlebotic Syndrome |
| Biliary Obstruction | Hernia | Post Radiation Changes |
| Bipolar Disorder | Home Care Issues | Pregnancy |
| Bite | Homicidal | Pregnancy Issue |
| Bizarre Behaviour | Hyperemesis Gravidarum | Presyncope |
| Bladder Problems | Hyperglycemia | Prostate Cancer |
| Borderline Personality Disorder | Hypersensitivity Pneumonitis | Pseudomyxoma Peritonei |
| Bradycardia | Hypertension | Pseudoseizure |
| Breast Mass | Hypertensive Emergency | Psychiatric Issue |
| Bronchiectasis | Hyperthyroid | Psychosis |
| Bronichtis | Hypervolemia | Pulmonary Embolism |
| Burn | Hypoglycemia | Pulmonary Fibrosis |
| Bursitis | Hypokalemia | Pyelonephritis |
| Cancer Pain | Hypomagnesemia | Rash |
| Cardiac Arrest | Hyponatremia | Reactive Airway Disease |
| Cast Check | Hypopituitarism | Renal Cancer |
| Cellulitis | Hypotension | Respiratory Failure |
| Cerebral Aneurysm | Hypovolemia | Respiratory Secretions |
| Chemical Exposure | Ill Defined Condition | Retinal Detachment |
| Chest Pain | Impulsive Behaviour | Sacral Ulcer |
| Chlostridium difficile | Infected Hematoma | Schizoaffective Disorder |
| Cholecystitis | Infection | Schizophrenia |
| Chronic Headache | Inflammation | Sciatica |
| Chronic Obstructive Pulmonary Disease | Inflammatory Bowel Disease | Scrotal Edema |
| Chronic Pain | INR Issue | Sebaceous Cyst |
| Chronic Pancreatitis | Insomnia | Seizure |
| Cirrhosis | Intracranial Hypertension | Self Harm |
| Cognitive Impairment | Ischemic Toe | Sepsis |
| Colitis | IV Access Issue | Sexual Health Education |
| Colon Cancer | IV Drug Use | Shock |
| Coma | Laceration | Shortness of Breath |
| Complex Regional Pain Syndrome | LAMA | Sickle Cell Crisis |
| Concussion | Large Bowel Obstruction | Sinusitis |
| Congestive Heart Failure | Leg Ulcer | Situational Crisis |
| Constipation | Liver Cyst | Sleep Apnea |
| Cough | Liver Failure | Small Bowel Obstruction |
| Crohn'sDisease | Loss of Consciousness | Social Issues |
| Decreased Level of Consciousness | LWBS | Spontaneous Bacterial Peritonitis |
| Deep Vein Thrombosis | Lymphadenitis | Stoma Issue |
| Dehydration | Lymphoma | Stridor |
| Delirium | Lymphoma | Stroke |
| Dementia | Malignancy | Subarachnoid Hemorrhage |
| Dental Pain | Malingering | Subconjunctival Hemorrhage |
| Depression | Mania | Subdural Hematoma |
| Dermatitis | Mastitis | Substance Misuse |
| Diabetes Issue | Mechanical Fall | Suicidal |
| Diabetic Ketoacidosis | Medical Device Problem | Supraventricular Tachycardia |
| Diabetic Neuropathy | Medical Note | Syncope |
| Diarrhea | Medication Issue | Tachycardia |
| Dislocation | Medication Reaction | Tempoal Arteritis |
| Diverticular Abscess | Medication Request | Thrombocytopenia |
| Diverticulitis | Melena | Thrombosis |
| Dizziness | Menorrhea | Transient Ischemic Attack |
| Dressing Change | Mental Health Issue | Temporomandibular Joint Pain |
| Duodenal Ulcer | Mental Retardation | Tonsillitis |
| Dysfunctional Uterine Bleeding | Metastatic Cancer | Tracheostomy Issue |
| Dysphagia | Migraine | Transfusion |
| Dystonia | Miscarriage | Trigeminal Neuralgia |
| Dysuria | Mood Disorder | Thrombotic Thrombocytopenic Purpura |
| Ear Pain | Motor Vehicle Collision | Upper Respiratory Tract Infection |
| Edema | Mucositis | Ureteric Stent Pain |
| Effusion | Multiple Complaints | Urethral Colic |
| Electrolyte Issue | Musculoskeletal Chest Pain | Urethral Erosion |
| Elevated Liver Enzymes | Musculoskeletal Pain | Urinary Issue |
| Endometriosis | Nausea & Vomiting | Urinary Retention |
| Enterocutaneous Fistula | Needle Stick Injury | Urinary Tract Infection |
| Epistaxis | Nephrostomy Tube Issue | Urosepsis |
| Esophageal Cancer | Neurology Issue Resolved | Vaginal Bleed |
| Esophagitis | Non Small Cell Lung Cancer | Vaginitis |
| Eyelid Issue | Numbness | Venous Stasis |
| Facial Trauma | Obstructive Sleep Apnea | Vertigo |
| Failure to Cope | Opioid Dependence | Violent behaviour |
| Fall | Osteomyelitis | Viral Illness |
| Febrile Neutropenia | Otitis Externa | Weaknessss |
| Fever | Otitis Media | Weight Loss |
| Fibroids | Ovarian Cyst | Wheelchair Malfunction |
| Fibromyalgia | Overdose | Withdrawal |
| Fistula Issue | Palpitations | Wound Issue |
| Flank Pain | Pancreatic Cancer |  |
| Foley Issue | Pancreatitis |  |
